# What Are We Worried About? Mid-life Couples’ Financial Concerns About Their Retirement

**DOI:** 10.1093/geroni/igab046.3292

**Published:** 2021-12-17

**Authors:** Jeremy Yorgason, Dikla Segel-Karpas, Ashley Ermer, Hailey Weller, Shenan Owens, Brandan Wheeler

**Affiliations:** 1 Brigham Young University, Provo, Utah, United States; 2 University of Haifa, Haifa, Hefa, Israel; 3 Montclair State University, Montclair, New Jersey, United States; 4 Brigham Young University--Provo, Provo, Utah, United States; 5 Brigham Young University, Brigham Young University - Provo, Utah, United States; 6 Mississippi State University, Mississippi State, Mississippi, United States

## Abstract

Retirement is an expected stage of life that couples plan for far in advance. Despite knowing that years of life without regular income are anticipated, some underprepare, leading to financial uncertainty in later years. In this study we explore financial concerns for retirement expressed by a sample of 335 midlife (Mage=44) couples that participated in the Flourishing Families study. We also examined predictors of those concerns across a 1-year period. Results suggested that both husbands and wives worried about insufficient income, excess spending, and heavy debt in retirement. Minor concerns included being worried about paying for their children’s education, net worth, and general expenses. Lower income was predictive of both husbands and wives being worried about having insufficient income in retirement. Higher income was predictive of husbands having concerns about excess spending. Although having retirement benefits was not predictive of any worries, having retirement savings was associated with wives having a greater likelihood of reporting worries about heavy debt and net worth in retirement. Better financial communication was associated with fewer husbands reporting concerns about excess spending and fewer wives reporting concerns about heavy debt. Having concerns about a spouse not being financially responsible were associated with more husbands reporting worries about excess spending and heavy debt in retirement. When wives reported higher social connection with a child, they also were more likely to report worries about expenses. Findings suggest that saving for retirement, communicating well about finances, and being financially responsible are associated with fewer financial concerns in retirement.

